# Alternative splicing and thermosensitive expression of *Dmrt1* during urogenital development in the painted turtle, *Chrysemys picta*

**DOI:** 10.7717/peerj.8639

**Published:** 2020-03-19

**Authors:** Beatriz Mizoguchi, Nicole Valenzuela

**Affiliations:** Department of Ecology, Evolution and Organismal Biology, Iowa State University, Ames, IA, United States of America

**Keywords:** Turtles, Spliceoforms, *Chrysemys picta*, *Dmrt1*, Sex determination, Sex differentiation, Transcriptional regulation evolution

## Abstract

**Background:**

The doublesex and mab-3 related transcription factor 1 (*Dmrt1*) is a highly conserved gene across numerous vertebrates and invertebrates in sequence and function. Small aminoacid changes in *Dmrt1* are associated with turnovers in sex determination in reptiles. *Dmrt1* is upregulated in males during gonadal development in many species, including the painted turtle, *Chrysemys picta*, a reptile with temperature-dependent sex determination (TSD). *Dmrt1* is reported to play different roles during sex determination and differentiation, yet whether these functions are controlled by distinct *Dmrt1* spliceoforms remains unclear. While *Dmrt1* isoforms have been characterized in various vertebrates, no study has investigated their existence in any turtle.

**Methods:**

We examine the painted turtle to identify novel *Dmrt1* isoforms that may be present during urogenital development using PCR, profile their expression by RNA-seq across five embryonic stages at male- and female-producing temperatures, and validate their expression pattern via qPCR with transcript-specific fluorescent probes.

**Results:**

A novel *Dmrt1* spliceoform was discovered for the first time in chelonians, lacking exons 2 and 3 (*Dmrt1* ΔEx2Ex3). *Dmrt1* canonical and ΔEx2Ex3 transcripts were differentialy expressed by temperature at stages 19 and 22 in developing gonads of painted turtles, after the onset of sex determination, and displayed a significant male-biased expression pattern. This transcriptional pattern differs from studies in other turtles and vertebrates that reported *Dmrt1* differential expression before or at the onset of sex determination. This study provides the first insight into *Dmrt1* transcriptional diversity in turtles and opens the door for future functional studies of the alternative *Dmrt1* transcript uncovered here.

**Conclusions:**

The discovery of an isoform in turtles indicate that alternative splicing may be a common feature of *Dmrt1* across vertebrates, as isoforms are also found in crocodilians, birds, mammals and fish, and this variation remains unexplained. The relatively late-onset of *Dmrt1* expression observed here contrasts with other turtles, indicating that *Dmrt1* is not the topmost male sex -determining factor in *C. picta*. When placed in a phylogenetic context, this discrepancy underscores the divergent regulation of *Dmrt1*, and of sexual development more generally, across vertebrates.

## Introduction

Sex determination is an important developmental process that contributes to the sexual fate of an individual. There are two extremes in the continuum of sex-determining mechanisms, one based on sexually dimorphic genomic content, called genotypic sex determination (GSD), and another on environmental factors, known as environmental sex determination (ESD) (Valenzuela, Adams & Janzen, 2003; Sarre, Georges & Quinn, 2004). Temperature-dependent sex determination (TSD) is the most common ESD mechanism in vertebrates, a polyphenism where individuals become male or female depending on the incubation temperature experienced by the developing embryo (Valenzuela & Lance, 2004; Bachtrog et al., 2014). TSD is present in many vertebrates, such as all crocodilians and tuatara, most turtles, some lizards and some fish (Valenzuela & Lance, 2004; Tree of Sex Consortium et al., 2014). Fewer examples are known of species with a mixed mechanism between GSD and TSD (Holleley et al., 2015; Yamamoto et al., 2014; Shine, Elphick & Donnellan, 2002) as some reports have been debunked (Mu et al., 2015; Valenzuela et al., 2014).

Many elements of the molecular network that regulate gonadal formation are shared between GSD and TSD species. In therian mammals, this process is triggered by *Sry* (sex-determining region of the Y chromosome), a gene that activates downstream genes of the male cascade (Eggers, Ohnesorg & Sinclair, 2014). All other vertebrates lack *Sry*, including TSD taxa, but non-Therians possess downstream genes in the male cascade, such as *Sox9*, *Wt1* and *Dmrt1* among others (Shoemaker & Crews, 2009) (Supplementary Material 1), some of which must be responsible for male determination and differentiation in the absence of *Sry*.

Within this molecular circuitry, an important component shared by vertebrates and invertebrates is *Dmrt1* (doublesex and mab-3 related transcription factor 1), a member of the *Dmrt* gene family which is mainly characterized by the presence of a zinc-finger DNA-binding domain, the DM domain (Raymond et al., 2000). This DM domain was first described in *Drosophila* (doublesex gene –*dsx*) and in *Caenorhabditis elegans* (mab-3 gene) (Volff et al., 2003). *Dmrt* genes are involved in sexual development, more specifically in male-specific differentiation, being expressed mainly in developing gonads. However, some *Dmrt* members might also participate in neural and muscular development (Hong, Park & Saint-Jeannet, 2007). *Dmrt1* is conserved at the nucleotide and protein sequence level, as well as in its function (Boyer et al., 2002; Bratus & Slota, 2006). *Dmrt1* also contains a ‘*male-specific domain*’ which is conserved across vertebrates and invertebrates (Jeong et al., 2009; Guan, Kobayashi & Nagahama, 2000), and is so called for its discovery in *Drosophila* where its sex-specific splicing produces dsx^m^ (Burtis & Baker, 1989; Burtis et al., 1991). The dsx^m^ isoform contains the male-specific region that binds to enhancers, promoting tissue-specific genes that repress genes related to female development (Coschigano & Wensink, 1993). A third important *Dmrt1* domain is the P/S-rich region, a non-DNA-binding domain involved in the transcription machinery, playing a role in the binding of enhancers and inhibitors that regulate mRNA production (Latchman, 1997). *Dmrt1* is not just an important element for vertebrate sexual differentiation, but in some vertebrates it is the top-most sex-determining gene. For instance, *Dmrt1* and its orthologs and paralogues were proposed as the male sex-determining gene in chicken (Smith et al., 2009), medaka fish (*dmy*) (Matsuda et al., 2002) and in the Chinese tongue sole (Cui et al., 2017; Herpin & Schartl, 2015). Notably, chicken *Dmrt1* is located in the Z-chromosome and works in a dosage manner (Teranishi et al., 2001). In contrast, the *Dmrt1*-paralogue *DM-W* is a female sex-determining gene in the frog *Xenopus laevis*, acting as an antagonist of *Dmrt1* expression (Yoshimoto et al., 2010). In contrast, in some species *Dmrt1* functions mainly in sexual differentiation rather than determination. For instance, in the rainbow trout, *rtDmrt1* is upregulated in males during testicular differentiation, and during spermatogenesis in adults (Marchand et al., 2000). In *Rana rugosa*, *Dmrt1* is also upregulated only during sex differentiation (Shibata, Takase & Nakamura, 2002). In the pejerrey, *Odontesthes bonariensis*, a fish with a mixed TSD+GSD system (Yamamoto et al., 2014; Hattori et al., 2012), *Dmrt1* is upregulated at male-producing temperatures (MPT) compared to female-producing temperatures (FPT) during gonadal development (Fernandino et al., 2008). Consistently, *Dmrt1* exhibits temperature-dependent expression in TSD reptiles, a lineage where the molecular evolution of *Dmrt1* is associated with turnovers in sex determination between TSD and GSD (Janes et al., 2014). Indeed, *Dmrt1* is upregulated under MPT starting at early embryonic stages in the TSD turtles *Trachemys scripta*, *Lepidochelys olivacea* and *Chelydra serpentina*, as well as the crocodile *Crocodylus palustris* (also TSD) (Kettlewell, Raymond & Zarkower, 2006; Torres Maldonado et al., 2002; Rhen et al., 2007; Anand et al., 2008). In *T. scripta* turtles and chicken, this early *Dmrt1* male-biased expression precedes the expression of other genes relevant for male development, including *Sox9*, suggesting that *Dmrt1* is upstream of *Sox9* in their sex determination cascade (Kettlewell, Raymond & Zarkower, 2006; Smith et al., 2009; Czerwinski et al., 2016). Furthermore, recent studies report that *Dmrt1* is required for male development in *T. scripta* (Ge et al., 2017) and in the GSD turtle *Pelosdiscus sinensis* (Sun et al., 2017). Thus, *Dmrt1* can act either in sex determination (deciding the sexual fate of the bipotential gonad) and in sex differentiation (contributing to the ensuing tissue development of the gonad) depending on its expression profile in particular species (Bratus & Slota, 2006).

So how does *Dmrt1* carry out these different roles? Genes may accomplish multiple functions by producing multiple isoform via alternative splicing (Anand et al., 2008), a process that allows a higher proteomic complexity without increasing genomic size. Some isoforms are important in sexual development. For instance, *Wt1* has 36 different reported isoforms, two of which (+KTS and –KTS) participate in sexual development (Bandiera et al., 2015). Each isoform encodes for a protein with a distinct DNA binding profile that differs in function, one being essential for male development and the other for survival of the gonadal primordium (Hammes et al., 2001). Because *Dmrt1* plays multiple roles in sexual development, including cell fate determination, postnatal Sertoli cells and primordial germ cells maintenance (Capriglione et al., 2010), an open question is whether those functions are undertaken by alternative *Dmrt1* isoforms. While *Dmrt1* isoforms have been reported in the TSD *Crocodylus palustris* (Anand et al., 2008) and in other vertebrates such as chicken, mice, zebrafish and rice field eel (Lu et al., 2007; Gao et al., 2005; Huang et al., 2005; Zhao et al., 2007b; Zhao et al., 2007a), no *Dmrt1* isoforms have been described in turtles.

Here we test the hypotheses that Dmrt1 isoforms exist in turtles and are differentially regulated by temperature, using the TSD painted turtle (*Chrysemys picta*) and leveraging RNA-seq data from a parallel study. Initial studies in *C. picta* profiled the *Dmrt1* expression in adrenal-kidney-gonad (AKG) complexes only (Valenzuela, 2010) without distinguishing between canonical transcripts and other isoforms that may exist, and no differential expression was detected. Later transcriptomic approaches (Radhakrishnan et al., 2017) revealed higher *Dmrt1* expression in developing testis during the thermosensitive period when gonads were analyzed separately, a pattern that had been masked by the adrenal-kidney (AK) expression when using AKGs (Valenzuela, 2010). These first transcriptomic data from painted turtles (Radhakrishnan et al., 2017) also revealed that RNA-seq is less sensitive than qPCR to detect subtler but significant differential expression, including at early stages of development for other important genes in the sexual development network (Supplementary material 1) such as *Wt1*, *Sf1*, *Dax1*, *Sox9*, and *Aromatase* (Valenzuela, Neuwald & Literman, 2013; Valenzuela, 2010; Valenzuela, LeClere & Shikano, 2006; Valenzuela & Shikano, 2007; Valenzuela, 2008a). Thus, here we (1) test for the presence of *Dmrt1* isoforms during the gonadal development of *C. picta*, and (2) test whether the canonical transcript or isoforms of *Dmrt1* exhibit differential expression by temperature consistent with *Dmrt1*’s role in sex determination or sexual differentiation in this TSD turtle using RNA-seq and qPCR validation.

## Methods

### Eggs collection, incubation, and tissue dissection

Freshly laid eggs were collected from a turtle farm and transported in moist vermiculite to the laboratory for incubation following standard protocols (Valenzuela, 2009). Specifically, eggs were cleaned from excess mud, marked with a unique ID, randomly assigned to boxes with moist sand (30 eggs per box), and placed in incubators at 26 °C (Male Producing Temperature—MPT) and 31 °C (Female Producing Temperature—FPT). Boxes were rotated daily in a clockwise fashion to control for potential temperature gradients within the incubators. Moisture inside the egg boxes was maintained constant by replacing evaporated water weekly. Embryonic development was monitored by egg candling. Embryos and tissues (detailed below) were dissected at stages before (stages 9 and 12), at the onset of (stage 15), in the middle of (stage 19), and at the end of (stage 22) the thermosensitive period (TSP) for *C. picta* (sensu (Yntema, 1968; Bull & Vogt, 1981)) and stored in RNA later (Invitrogen) at −20 °C until processing. All samples were treated identically.

### RNA extraction and cDNA conversion

RNA was extracted from trunks of stage 9 embryos when the gonadal primordium cannot be separated (*n* = 15 per T° per replicate), adrenal-kidney-gonad (AKG) complex of stage 12 embryos (when genital ridge may be present in *C. picta* given that urogenital tissue is present by this stage in *T. scripta* (Spotila, Spotila & Hall, 1998), from AKG of stage 15 embryos (*n* = 15 per T° per replicate) (when bipotential gonads could not be separated from AK), and from separated gonads from stage 19 (*n* = 13 per T° per replicate) and stage 22 (*n* = 12 per T° per replicate) embryos using Qiagen Rneasy™ Mini (stages 9, 12 and 15) and Micro (stages 19 and 22) kits, following the manufacturer’s instructions. RNA was quantified using a NanoDrop Spectrophotomoter and RNA quality was assessed by the presence of ribosomal bands in 1% agarose gels. Extracted RNA was stored at −80 °C until processing. All RNA was extracted from individual embryos and 200 ng to 1 µg of RNA was retro-transcribed to cDNA by RT-PCR using Invitrogen SuperScript™ VILO™ Synthesis kit following the manufacturer’s instructions. cDNA was stored at −20 °C.

### Identification of *Dmrt1* isoforms by PCR and Sanger sequencing

Primers (Dmrt1-F: 5′ CTT GTT AGC CGA ACC TCT CT 3′ and Dmrt1-R: 5′ AGA ATG CAC TTG ATC TCC TG 3′) were designed at the untranslated region (UTR) of the *Dmrt1* gene, based on the *C. picta* genome (Badenhorst et al., 2015; Shaffer et al., 2013) using Geneious (Kearse et al., 2012). PCR amplification of *Dmrt1* transcripts used 1 µL of pooled cDNA (from all stages and temperatures) as template in 15 µL reactions containing 1X Taq buffer, 1.5 mM MgCl_2_, 0.2 mM dNTPs, 0.4 µM of each primer (Dmrt1-F and Dmrt1-R), 0.4U Taq polymerase and 10.5 µL water. PCR conditions included an initial denaturing step at 94 °C for 3 min, followed by 35 cycles of denaturing at 94 °C for 30 s, annealing at 58 °C for 30 s, and extension at 72 °C for 90 s. Amplicons were visualized in 0.8% agarose gel stained with EtBr, and their size estimated using a 1 kb plus ladder (Invitrogen). Amplicon bands were cut from the agarose gel, placed in 50 µL distilled water, incubated 5 min at 65 °C, 10 min at −80 °C and then centrifuged 10 min at 4 °C, after which 1 µL was used as template in a secondary 50 µL PCR reaction using an annealing temperature of 61 °C. PCR products from reactions that yielded a single amplicon were cleaned using Ampure beads, and Sanger sequenced. DNA sequences were analyzed in Geneious (Kearse et al., 2012) to assess their quality, aligned by BLAST to the *C. picta* genome assembly 3.0.3 (Badenhorst et al., 2015) for annotation and to assess the similarity of any isoforms to the canonical *Dmrt1* transcript.

### Identification and expression profiling of *Dmrt1* isoforms from RNA-seq data

#### Differential expression analysis of RNA-Seq data

To complement the identification of isoforms by PCR, we leverage RNA-seq data obtained in duplicate for a parallel study from *C. picta* embryos at five developmental stages, incubated at 26 °C and 31 °C (MPT and FPT, respectively) (see Data Availability). Total RNA was extracted as described above and 20 duplicate mRNA libraries from the same 5 stages and 2 temperatures were constructed using 1 µg total RNA pooled from 11–15 embryos (equal RNA amount per embryo) per stage per temperature per replicate, using the KAPA Stranded mRNA-seq kit (KK8421). Libraries were sequenced using Illumina’s HiSeq 4000 protocol, which generated 50 million 150 bp paired-end cleaned reads per library on average.

The bioinformatics pipeline employed here is illustrated in Supplementary Material 2A and scripts are provided in Supplementary material 3. We used the *Chrysemys picta* genome version 3.0.3 (NCBI) (Badenhorst et al., 2015) as reference genome. Initial quality control of raw reads was carried out with FASTQC (Andrews, 2010), followed by removal of adapters and low-quality reads using Trimmomatic (Bolger, Lohse & Usadel, 2014). Trinity (Haas et al., 2013) was used for read normalization. We used HISAT2 (Kim, Langmead & Salzberg, 2015) to map the reads to the *C. picta* reference genome. Mapping was conducted separately by stage and temperature, with 2 replicates each. All steps of the RNA-seq analysis were run at the High Performance Computing facility from Iowa State University.

Then we used Samtools view tool (Li et al., 2009) to select the reads that were uniquely mapped to the reference genome to avoid false positives and enhance the accuracy during the assembly of transcripts. Gene annotations were retrieved from NCBI and used as input along with the uniquely mapped and normalized reads, to assemble the transcripts using StringTie (Pertea et al., 2016). For the differential expression analysis, we pooled together all transcripts from all stages and temperatures produced by StringTie, to assemble an overall transcriptome using the StringTie merge tool.

Read counts were quantified with Kallisto (Bray et al., 2016) (Supplementary Material 4) using the transcriptome generated by StringTie and the normalized reads as input. We used DESEQ2 to assess differential expression based on the read counts, and significance was assessed at an alpha of 0.05.

Because not all *Dmrt1* transcripts that were detected by PCR and Sanger sequencing were present in the transcriptome assembled by using the whole genome as a reference, we developed an alternative sub-genomic approach as follows. First, we extracted the scaffolds assigned to chromosome 6 of the *C. picta* 3.0.3 genome where *Dmrt1* is located, plus all the unplaced scaffolds containing the other *Dmrt* members. We refer to this reference sub-genome as CPI-6U hereafter. This CPI-6U ‘reference sub-genome’ was then used to map the reads and assemble *Dmrt1* transcripts. We followed the same steps as with the whole genome approach and compared the results. The results were robust to using the Tuxedo pipeline (Trapnell, Pachter & Salzberg, 2009; Trapnell et al., 2012).

#### Validation of Dmrt1 isoform identification by splice junction analysis

Additionally, in a third approach, we tested for the presence of all potential *Dmrt* family isoforms in our RNAseq data by examining the mapping of the RNA-seq reads to all splice junctions of all *Dmrt* genes using Geneious (Kearse et al., 2012) (Supplementary Material 2B), in order to avoid misidentifying other *Dmrt* transcripts as *Dmrt1* isoforms . First, we mapped reads to a reference containing all *Dmrt* gene regions (first mapping step), and retained all reads that mapped to *Dmrt* genes and exons junctions to produce a reduced read dataset that was then mapped to a second reference file containing all possible exon junction combinations for each *Dmrt* gene. Results were inspected manually to identify any reads mapped to junctions undetected in previous steps and which would indicate the existence of additional isoforms in our RNA-seq data.

#### qPCR validation

We validated our RNA-seq data results further by using qPCR and Taqman probes, which also permitted us to profile transcript-specific expression during embryonic development at MPT and FPT in individual embryos. Primers and Taqman probes were designed for each *Dmrt1* isoform (Table 1), as well as for ß-actin, a housekeeping gene used for normalization of gene expression by qPCR in previous studies of *C. picta* (Valenzuela, Neuwald & Literman, 2013), and whose expression was steady between temperatures and across stages in our study as determined by ANOVA (Supplementary Material 5). Expression data by qPCR was obtained from 12-15 embryos per temperature (MPT and FPT) per stage (these embryos were different from the embryos used for RNA sequencing). *Dmrt1* Taqman probes were designed to match unique exon junctions of each isoform, in order to profile isoform-specific expression. qPCR was carried out in an Mx3000P real time PCR thermal cycler (Stratagene) using IDT Prime Time Gene Expression Mastermix. This mastermix already contains the DNA polymerase, dNTPs, MgCl_2_, enhancers and stabilizers, in concentrations undisclosed by the manufacturer. Optimization was run for individual genes using 6 different concentrations of primers (100 nM, 200 nM, 300 nM, 400 nM, 500 nM, 600 nM) and a fixed concentration of probe (250 nM) followed by probe optimization, with 6 different concentrations of probes (100 nM, 150 nM, 200 nM, 250 nM, 300 nM, 350 nM) combined with the optimal primer concentration of each gene. Later, multiplex optimization reactions with optimal primers and probes concentrations were tested, containing 5 uL of commercial mastermix, *ß-actin* forward and reverse primers, *ß-actin* Taqman probe, *Dmrt1* forward and reverse primers for either the canonical or the non-canonical isoform and the corresponding Taqman probe, 2 uL of cDNA and water to 15 ul. Thermal profile was 95 °C for 3min followed by 50 cycles of 95 °C for 30s and 60 °C for 1min. All reactions were run in duplicate. A standard curve was generated for each transcript by pooling RNA from all samples (100 ng per individual), and then diluting the pooled RNA using 1:5 ratio, to obtain a total of eight standards. Standards were included in duplicate in each qPCR plate. Standard curves were used to calculate qPCR efficiency and R^2^ values.

**Table 1:** Primer and Taqman probe sequences used for multiplex qPCR and optimal ß-actin concentrations.

Transcript	Sequences	Optimal *ß-actin* concentration
*ß-actin*	Forward: 5′ TGTGCTGCTTACAGAGG 3′ Reverse: 5′ GTACGACCAGAGGCCTA 3′ Probe: 5′/CY5/GCCAACAGAGAAAAGATGACACAGATC 3′	500 nM (c)/200 nM (i) 500 nM (c)/200 nM (i) 250 nM (c)/100 nM (i)
*Dmrt1* canonical	Forward: 5′ CCAACACATTCAACAAACA 3′ Reverse: 5′ ACTGCTGTAGTAGGTGGAGTC 3′ Probe: 5′/FAM/ATCAGAGGGACGGATGCTCATTCAG 3′	500 nM 500 nM 300 nM
*Dmrt1* isoform	Forward: 5′ TACTCCTCGCCACTGAA 3′ Reverse: 5′ CACTCTGGCCCAGGTAG 3′ Probe: 5′/FAM/TGGCAGCCAGATGAAAAGCACAG 3′	600 nM 600 nM 300 nM

**Notes.**

(c) ß-actin concentration for PCR of *Dmrt1* canonical transcript (i) ß-actin concentration for PCR of non-canonical isoform

### Data analysis

Samples with Cq values deviation >0.5 were discarded from further analysis. All samples displayed coefficient of variation <10% between technical replicates and were thus included in the analyses. Relative expression was calculated using the Pfaffl method (Pfaffl, 2001) (which takes into account differences in reaction efficiencies to calculate relative expression values), with ß-actin as the reference gene for normalization. An ANOVA was used to test for differences in *Dmrt1* expression between temperatures across stages, and post hoc t-tests were then used to identify the stages at which differential expression was significant (at an alpha of 0.05).

## Results

Here, we investigated the transcriptional dynamics of the *Dmrt1* gene, in terms of its alternative splicing and thermosensitive transcription in the TSD turtle *Chrysemys picta*. First, we investigated whether *Dmrt1* produces alternative spliceoforms to the canonical transcript, and second, we profiled the expression of the identified transcripts, to build working hypotheses about the potential role of *Dmrt1* in the sexual development of *C. picta* that could guide future functional assays.

### Alternative splicing

**Figure 1:**
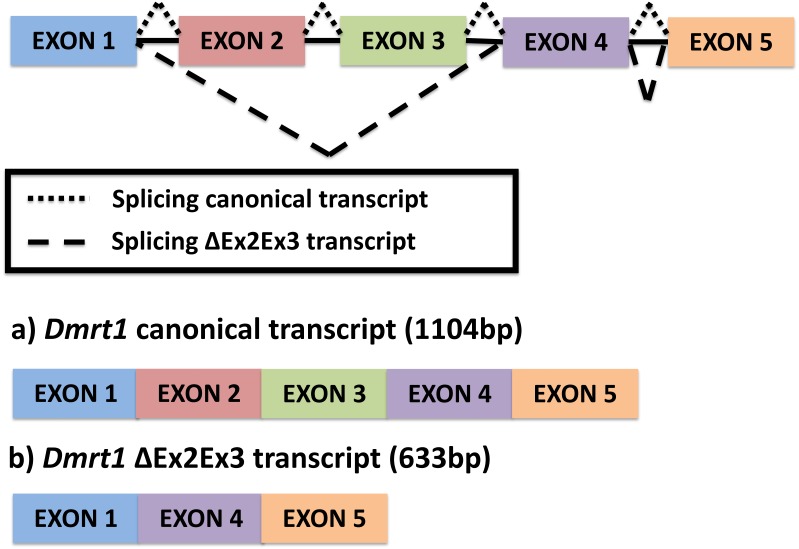
Alternative splicing of *Dmrt1* in *Chrysemys picta* turtles discovered in the present study.

A single novel isoform was detected by PCR amplification using primers at the UTR region of the *Dmrt1* gene sequence, followed by Sanger sequencing. In contrast to the canonical *Dmrt1* transcript that contains all 5 exons of the *Dmrt1* gene, the novel *Dmrt1* isoform lacks exons 2 and 3 (Fig. 1) (hereafter referred to as *Dmrt1* ΔEx2Ex3). Likewise, our mapping of RNA-seq reads to the full set of potential junctions of all exon pairs for *Dmrt1* (and for all other *Dmrt* genes in the painted turtle genome) detected this unique alternative spliceoform exclusively and no other alternative *Dmrt1* transcript.

The full canonical *Dmrt1* (hereafter referred to as *Dmrt1*) cDNA sequence is 1104 bp long. This canonical *Dmrt1* transcript encodes a protein containing the three characteristic domains that are conserved in the *Dmrt1* of other vertebrates, namely, the DM domain, the male-specific domain, and the proline- (P-) and serine-(S-)rich regions (Adolfi et al., 2015). On the other hand, the novel *Dmrt1* ΔEx2Ex3 cDNA is 633 bp long, and lacks the male-specific domain and the P- and S-rich region. Both transcripts were detected by qPCR at all stages and tissues examined, but their expression was accentuated at MPT at stage 19 in the individual gonads, as described below.

### Dmrt1 expression profiling by RNA-seq

**Figure 2:**
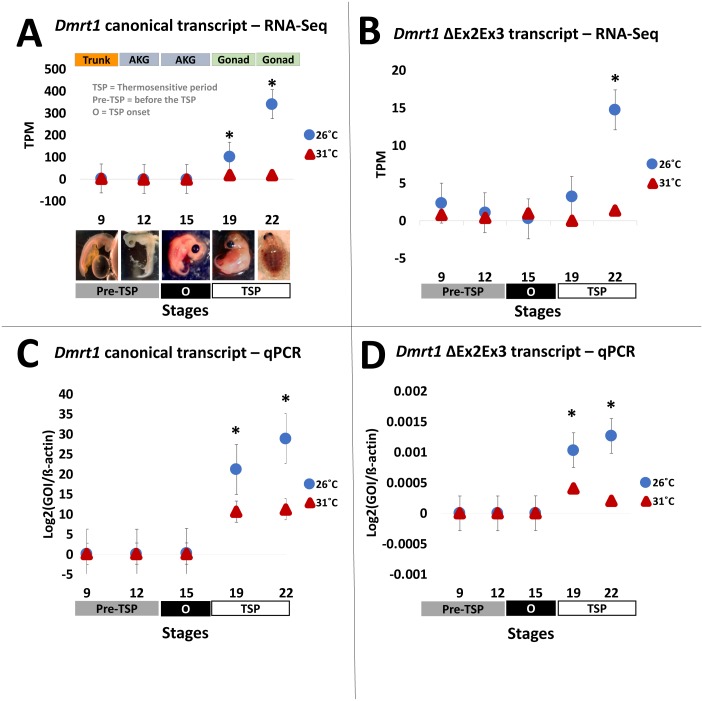
Transcription of *Dmrt1* spliceoforms during embryonic development of *Chrysemys picta* turtles. Averge expression level of canonical and *Dmrt1* ΔEx2Ex3 transcripts assessed by RNA-Seq (A,B) and by qPCR using TaqMan probes (C,D). Bars represent standard deviations. *, significant differences in expression between male- and female-producing temperatures (*p* < 0.05).

Results from our quantification analysis using duplicate RNA-seq experiments, indicate that the canonical *Dmrt1* transcript in the painted turtle exhibits very low and monomorphic expression before the thermosensitive period in the embryonic trunks (stage 9) and AKGs (stages 12 and 15), but it becomes significantly upregulated under MPT in the gonads alone at stages 19 and 22 (Figs. 2A–2B), showing maximal expression towards the end of the thermosensitive period (stage 22). However, the *Dmrt1* ΔEx2Ex3 transcript was not detected in RNA-Seq analysis at any stage and temperature when using the whole genome analysis approach. However, when using the CPI-6U reference sub-genome, the *Dmrt1* ΔEx2Ex3 transcript was assembled successfully. The sub-genome approach revealed that Dmrt1 ΔEx2Ex3 has quite a similar expression pattern to the canonical *Dmrt1* transcript, with monomorphic expression before the thermosensitive period followed by significant differential expression at MPT at stage 22. However, expression levels of *Dmrt1* ΔEx2Ex3 were orders of magnitude lower compared to the canonical transcript. Expression levels for ß-actin, used as the normalizer gene, did not differ significantly across stages and temperature treatments (Supplementary Material 5) as accessed by an ANOVA (*F* = 14.76, *p* = 0.52).

### qPCR validation

All qPCR reactions had standard curves with an R^2^>0.96 and all negative controls yielded no amplification. The qPCR results mimicked the RNA-seq results obtained for the canonical *Dmrt1* transcript. Namely, *Dmrt1* was upregulated in embryos incubated at MPT compared to FPT, a pattern that became significant at stage 19 and was accentuated at stage 22 (Figs. 2C–2D). qPCR results revealed that the abundance of the *Dmrt1* ΔEx2Ex3 transcript is an order of magnitude lower than for the *Dmrt1* canonical transcript, although ΔEx2Ex3 is also upregulated at MPT compared to FTP at stage 19 and 22 (whereas the CPI-6U RNA-Seq analysis detected ΔEx2Ex3 upregulation at MPT only at stage 22).

## Discussion

**Figure 3:**
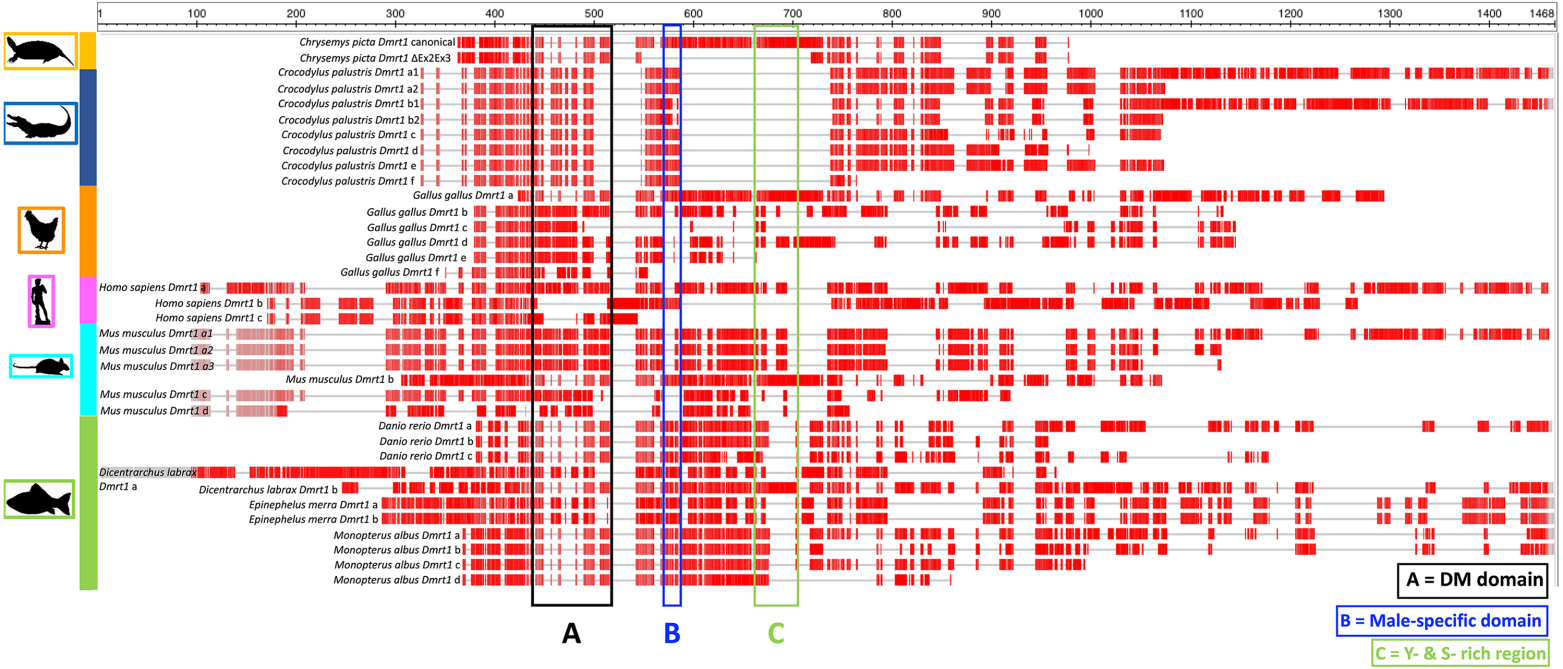
Protein alignment of canonical and ΔEx2Ex3 *Dmrt1* transcripts in *Chrysemys picta* and isoforms from selected vertebrates. Protein sequences from the canonical and *Dmrt1* isoforms correspond to those listed in Table 2. Red blocks illustrate conserved regions, gray horizontal lines illustrate sequence gaps. Colored vertical boxes denote *Dmrt1* domains. See Supplementary Material 6 for full alignment.

In this study we identified a novel *Dmrt1* spliceoform in *Chrysemys picta*, a TSD turtle, the first report of a *Dmrt1* isoform in any turtle irrespective of their sex-determining mechanism. *C. picta*’s novel isoform is unique in its sequence compared to the *Dmrt1* isoforms that have been identified in other vertebrates including fish, crocodilians, birds and mammals (Table 2, Fig. 3). While most *Dmrt1* non-canonical transcripts described to date in *C. picta* and other species retain the highly conserved DM domain (an exception is found in mouse (Lu et al., 2007)), such conservation is not preserved in other domains as described below. Namely, compared to the canonical *Dmrt1* transcript, *C. picta*’s novel *Dmrt1* ΔEx2Ex3 isoform retains exon 1 (where the DM domain is located) but it differs at the 3′ end downstream the DM domain where it lacks exons 2 and 3. Changes at the 3′ region downstream of the DM domain are also observed in three different *Dmrt1* isoforms in zebrafish (Guo et al., 2005), in the Indian mugger, a TSD crocodilian that produces eight *Dmrt1* isoforms (Anand et al., 2008), and in chicken whose *Dmrt1* generates six isoforms (Anand et al., 2008; Zhao et al., 2007a). On the other hand, the European sea bass produces two *Dmrt1* isoforms that differ by a 78 bp insertion, creating a separation between the Tyrosine (Y)- and the Serine (S)-rich domains (Deloffre et al., 2009), whereas the mouse generates *Dmrt1* isoforms that either lack the Y-rich region, lack both Y- and S-rich regions, or lack the DM domain (Lu et al., 2007). The Y- and S-rich domain differs from the P- and S- domain, and are not encountered in all vertebrates that encode *Dmrt1*. The specific role of the Y- and S-rich domain remains unknown, but some speculate that it may be important for the DM domain protein dimerization (Deloffre et al., 2009). Other vertebrate *Dmrt1* isoforms from various taxa analyzed in this study also differ in the presence or absence of the male-specific domain and/or the P- and S-rich region (that are present in exons 2 and 3 of *C. picta*) (Fig. 3). These isoforms are all male-specific (except for chicken *Dmrt1c*), and their expression was also lower compared to their respective canonical transcripts. Besides the presence/absence of characteristic domains, the transcription splice sites vary, leading to the formation of isoforms of varying length according to the number of exons that are included/excluded. There is no consensus about the specific function of the diversity of *Dmrt1* isoforms, and their expression profiles in other vertebrates led to the still untested hypothesis that they could be involved in regulating the canonical transcript, acting as coregulatory factors, and thus mediating sexual development (Lu et al., 2007; Anand et al., 2008).

**Table 2:** Number of *Dmrt1* isoforms reported to date in vertebrates.

**Group**	**Species**	**# of isoforms**	**Source**
Fish	Zebrafish	3	Guo et al. (2005)
Fish	European sea bass	2	Deloffre et al. (2009)
Fish	Honeycomb grouper	2	Alam et al. (2008)
Fish	Rice field eel	4	Huang et al. (2005)
Turtle	Painted turtle	2	This study
Crocodilians	Indian mugger	8	Anand et al. (2008)
Birds	Chicken	6	Zhao et al. (2007b)
Mammals	Mouse	4	Lu et al. (2007)
Mammals	Human	3	Cheng et al. (2006)

Our expression profiling revealed the transcriptional dynamics of the novel turtle isoform. *C. picta*’s *Dmrt1* ΔEx2Ex3 transcript was upregulated at stages 19 and 22 at MPT, but its transcription was an order of magnitude lower compared to the canonical transcript. This is consistent with most *Dmrt1* isoforms described in vertebrates which also display male-biased expression and lower expression compared to the canonical transcript (Lu et al., 2007; Guo et al., 2005; Anand et al., 2008; Deloffre et al., 2009; Zhao et al., 2007a). An exception is *Dmrt1c* in chicken, which is upregulated in females at stage 31 of embryonic development, which corresponds to the time of gonadal differentiation (Zhao et al., 2007a). It has been hypothesized that isoforms derived from alternative splicing might act as transcriptional regulator of the canonical transcript by affecting access to its activators or repressors (Lu et al., 2007; Anand et al., 2008). It is unclear whether the novel *Dmrt1* ΔEx2Ex3 isoform identified in *C. picta* plays a functional role despite its low transcription level as occurs for other genes in other taxa. For instance, differences in isoform abundance of glucocorticoid receptor (GR), some of which are expressed at very low levels, are linked to immune thrombocytopenia (ITP) (Hamed et al., 2015; Ma et al., 2013). In particular, the transcription of isoform GRß is extremely low compared to the isoform GR*α*, such that GRß protein is undetectable (Ma et al., 2013). However, GRß mRNA transcript works as a regulator of GR*α* mRNA transcript activity, and disruption of the GR*α*/GRß ratio may be related to resistance to glucocorticoids in ITP (Hamed et al., 2015), as this ratio predicts how the cell will respond to glucocorticoid treatments. Our data show that the *Dmrt1* canonical/ΔEx2Ex3 ratio responds to temperature in stage 15 AKGs of *C. picta* (Table 3), which corresponds with the onset of the TSP, a critical time for sex determination (though the expression of each individual isoform did not vary significantly by temperature at stage 15). However, the hypothesis that the *Dmrt1* canonical/ΔEx2Ex3 ratio plays an important role in the sexual development of *C. picta* requires functional testing, as well as isolation of gonadal from AK expression before stage 19. Also unknown is whether *Dmrt1* thermosensitive transcription in *C. picta* is epigenetically regulated as it is in the TSD turtle *T. scripta* (Ge et al., 2018).

**Table 3:** Ratio of *Chrysemys picta’s Dmrt1* transcripts (canonical/ΔEx2Ex3) at the embryonic stages studied.

	**Stage 9**	**Stage 12**	**Stage 15**	**Stage 19**	**Stage 22**
**26 °C**	278428.7	278428.7	106777.1	20465.8	22812.3
**31 °C**	28134.7	119667.9	99133.6	26103.9	54215.8
***p*-value**	0.4359	0.2416	0.02362	0.0767	0.2464

**Notes.**

Statistical significance between temperatures was assessed by *t*-tests with alpha = 0.05.

Our results also provide important novel insights into the expression of the canonical transcript of *Dmrt1* in *C. picta*, which was upregulated under MPT at stages 19 and 22. These results corroborate the expression pattern of *Dmrt1* observed in *C. picta* gonads in a previous transcriptomic analysis that used a single replicate (Radhakrishnan et al., 2017) providing a much needed validation. These observations contrast with previous qPCR studies (Valenzuela, 2010) that failed to detect significant differential expression of *Dmrt1* between individuals incubated at MPT and FPT at the same developmental stages examined here. The discrepancies are likely due to technical differences between studies. Specifically, in the earlier study (Valenzuela, 2010) expression at stages 19 and 22 was profiled in AKG complexes such that the expression from AK tissue likely masked the expression from gonadal tissue alone, thus obscuring the significant differences between temperatures that we detected here and in Radhakrishnan et al. (2017). The same masking phenomenon was reported in *C. picta* and other turtles for a number of other genes (Valenzuela, Neuwald & Literman, 2013; Ramsey & Crews, 2007). Likewise, care should be taken when interpreting our results, since extra gonadal tissues were included in our analysis at stage 9 (trunks) and at stages 12 and 15 (AKGs) due to the difficulty of isolating the embryonic gonads at these time points, such that *Dmrt1* expression in the genital ridge or bipotential gonad could be confounded with extra-gonadal expression.

**Figure 4:**
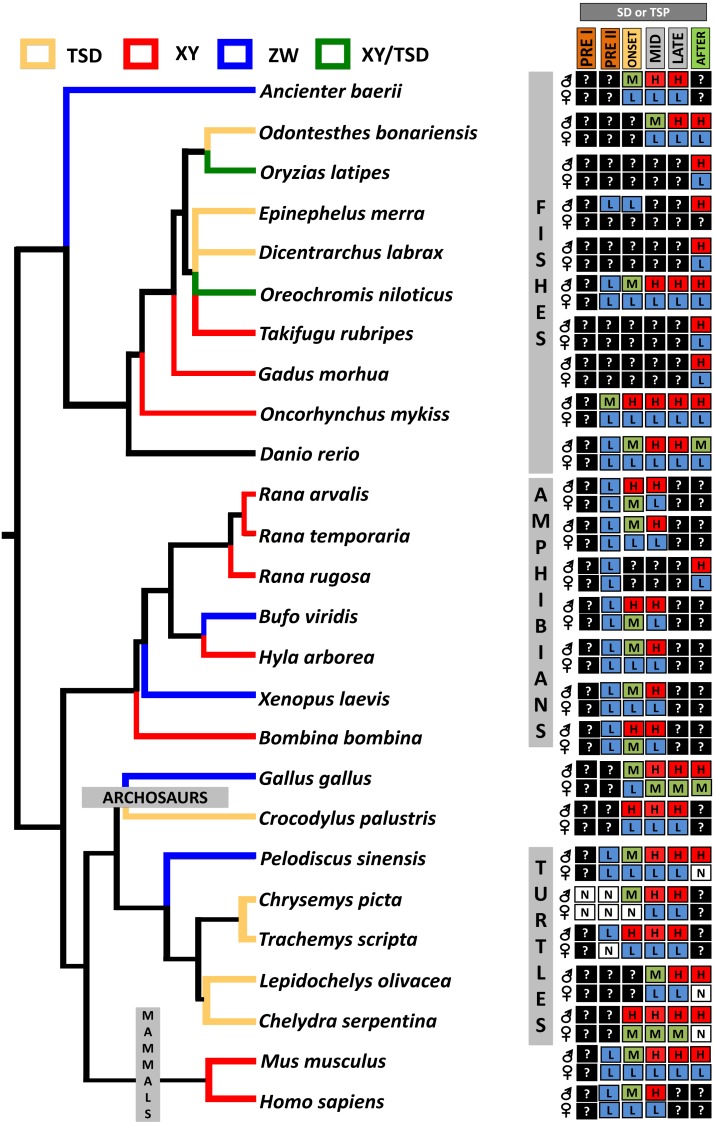
Transcriptional patterns of *Dmrt1* during embryonic development of selected vertebrates with different sex-determining mechanisms. Expression levels are color-coded as: none detected (white, N), low (blue, L), medium (green, M), high (red, H) transcription, and not studied (black, ?). Stages examined correspond to those before, during and after the thermosensitive period (TSP) for TSD taxa or sex differentiation (SD) for GSD taxa, following Valenzuela, Neuwald & Literman, (2013).

From an evolutionary perspective, it has been hypothesized that the ancestral function of *Dmrt1* was to trigger male sexual development and that new functions expanded later to regulate the development of sexual dimorphism (Matson & Zarkower, 2012). This conservation in function of *Dmrt1* in male development is reflected in its sexually dimorphic expression pattern across vertebrates, as observed in isolated gonadal tissue in *C. picta* (this study), and in other TSD and GSD reptiles, such as *T. scripta, Pelodiscus sinensis, Lepidochelys olivacea, Chelydra serpentina* and *Crocodylus palustris* (Kettlewell, Raymond & Zarkower, 2006; Sun et al., 2017; Torres Maldonado et al., 2002; Rhen et al., 2007; Ge et al., 2017). However, differences exist in the timing of *Dmrt1* expression among species (Fig. 4), reflecting the divergence of its placement in the sex determination/differentiation regulatory network. Indeed, various studies demonstrated *Dmrt1* upregulation in developing males (at MPT, or in XY or ZZ male individuals) in most vertebrates investigated to date (Fig. 4). *Dmrt1* (and homologs) is required to trigger the male sex determination pathway in the Chinese tongue sole, medaka and chicken (Cui et al., 2017; Nanda et al., 2002; Smith et al., 2009). In other species of fish, amphibians and in mammals, *Dmrt1* plays a pivotal role in Sertoli and germ cells differentiation and testicular maintenance, being upregulated in males during the mid or late stages of sex determination or after the sex determination period (Raymond et al., 1999; Jørgensen et al., 2008; Deloffre et al., 2009; Fernandino, Guilgur & Somoza, 2006; Kobayashi et al., 2008; Johnsen et al., 2010; Yamaguchi et al., 2006).

In turtles, functional assays demonstrated that *Dmrt1* is required for male development in *T. scripta* and *P. sinensis* and it sits upstream of *Sox9* and *AMH* in the male sexual development cascade (Supplementary Material 1), two important genes for testis development that are upregulated after *Dmrt1* in these two species (Ge et al., 2017; Sun et al., 2017). Likewise, *L. olivacea* and *C. serpentina* exhibit *Dmrt1* upregulation prior to *Sox9* (Rhen et al., 2007; Torres Maldonado et al., 2002). In contrast, our RNA-seq and qPCR results detected *Dmrt1* upregulation at MPT during the thermosensistive period, at the same stages (19 and 22) at which *AMH* is differentially expressed (Radhakrishnan et al., 2017), whereas the male differentiation genes *Sox9, Wt1* and *Sf1* are upregulated at MPT before *Dmrt1* (stage 15) (Valenzuela, Neuwald & Literman, 2013; Radhakrishnan et al., 2017). Again, we note that AKGs were examined at stage 15, and the extragonadal tissue could confound gonadal expression. However, studies in stage 15 *T. scripta* embryos detected no *Dmrt1* expression in AKs (Shoemaker et al., 2007), qualitatively upregulation at MPT in AKGs (Kettlewell, Raymond & Zarkower, 2000), but not quantitative differences in AKGs (Murdock & Wibbels, 2006) or isolated gonads (Shoemaker et al., 2007; Czerwinski et al., 2016). Given that *C. picta* and *T. scripta* are closely related species, and assuming that stage 15 AK expression is also low/absent in *C. picta*, the monomorphic expression we observe in AKGs at stage 15 may reflect actual monomorphic *Dmrt1* gonadal expression. Combined, current data indicate that *Dmrt1* is not the topmost trigger in the male sexual development cascade in *C. picta*. However, there is strong evidence suggesting that *Dmrt1* contributes to male sexual development in *C. picta*, given its upregulation at MPT during the TSP and its predominantly conserved function across other taxa. Functional studies are needed to elucidate *Dmrt1*’s specific role in painted turtles.

We note the disparity of results between the two approaches of RNA-Seq analysis. The use of a subgenome allowed us to assemble the novel transcript identified by PCR and Sanger sequencing, but the same did not happen when using the whole genome as reference for mapping the reads. We believe that using the whole genome contributes to (1) discard transcripts of extremely low expression, as most assembler software packages consider them as “not true” and (2) using a genome-guided assembly can be restrictive in assembling low-expression isoforms. Also, we first identified the *Dmrt1* ΔEx2Ex3 transcript using PCR and sequencing and we were able to detect this transcript using probe-based qPCR, using a probe specific of *Dmrt1* ΔEx2Ex3 transcript.

Lastly, we performed qPCR in numerous biological replicates to validate the biological conclusions derived from previous RNA-seq data (Radhakrishnan et al., 2017). Earlier qPCR studies of *C. picta* (Valenzuela, LeClere & Shikano, 2006; Valenzuela, 2008b; Valenzuela, 2010) detected differential expression for *Sox9, Wt1* and *Sf1* at stages preceding those when RNA-Seq analysis detects upregulation (Radhakrishnan et al., 2017), which is not surprising given the higher sensitivity of qPCR to detect subtle differences that occur at these earlier stages in *C. picta*. The discordance between qPCR and RNA-seq analysis we observe is not unique. For instance, some studies on oysters found that around 55% (19 out 34) of the results would match between RNA-seq and qPCR (Shi & He, 2014), while other studies on bacteria observed high correlation between RNA-seq and qPCR expression results (Camarena et al., 2010).

## Conclusion

Here we identified the first non-canonical *Dmrt1* isoform (*Dmrt1* ΔEx2Ex3) in any turtle species, and elucidated the response to temperature of the two *Dmrt1* transcripts of *C. picta*. Our data revealed that both *Dmrt1* transcripts are upregulated at MPT during the TSP in isolated gonads, consistent with previous studies on canonical *Dmrt1* expression, and supporting the notion that *Dmrt1* may be a fundamental element for male sexual development in painted turtles. Monomorphic *Dmrt1* expression was observed at earlier developmental stages when extragonadal tissues were included that could have confounded *Dmrt1* expression from the genital ridge or early bipotential gonad. Nonetheless, compared to *T. scripta*, our data from stage 15 *C. picta* suggest that *Dmrt1* is unlikely to sit at the top of the male determination cascade in painted turtles, although further research is warranted, particularly in light of the thermosensitive response of the ratio of the canonical transcript to *Dmrt1* ΔEx2Ex3 at stage 15. Additional differences in the timing of expression among taxa provide evidence of a divergent regulation of *Dmrt1* across vertebrates irrespective of the mode of sex determination (TSD or GSD), unlike *Dmrt1*’s molecular evolution, which appears linked to transitions between TSD and GSD in terms of the rate at which sequences evolve (Literman et al., 2018) and in terms of shifts in the sequence of amino acids (Janes et al., 2014). Why exactly does *Dmrt1* vary in the number of isoforms that are produced in different taxa remains an open question. More broadly, our results contribute to ongoing efforts to characterize isoforms in genetic networks, which are often neglected. Given the role of alternative splicing to increase transcriptomic diversity without demanding greater genomic complexity, it is critical to investigate the presence of isoforms and their function using a combination of approaches as shown here, if we are to gain a comprehensive picture of embryonic development.

##  Supplemental Information

10.7717/peerj.8639/supp-1 Supplemental Information 1 Partial vertebrate gene-regulatory network underlying gonadal formation *Sry* is present only in Therian mammals. Modified from (Mizoguchi & Valenzuela, 2016)

10.7717/peerj.8639/supp-2 Supplemental Information 2 (A) RNA-seq analysis workflowused to quantify *Dmrt1* expression in *Chrysemys picta* (B) Junction analysis workflow

10.7717/peerj.8639/supp-3 Supplemental Information 3 Scripts used in this study for bioinformatics analyses

10.7717/peerj.8639/supp-4 Supplemental Information 4 Read counts obtained from Kallisto for expression analyses Reads are separated by developmental stage and incubation temperature (26 °C = Male Producing Temperature, 31 °C = Female Producing Temperature).

10.7717/peerj.8639/supp-5 Supplemental Information 5 Gene expression of ß-*actin* transcript during embryonic stages of *Chrysemys picta* Panel A illustrates RNA-Seq results. Panel B illustrates TaqMan qPCR results. Bars represent standard deviations.

10.7717/peerj.8639/supp-6 Supplemental Information 6 Full alignment of *Dmrt1* isoforms of vertebrates mentioned in Table 2

## Additional Information and Declarations

### Data Availability

The following information was supplied regarding data availability: The RNAseq data is available in NCBI: study number SRP099173, bioproject PRJNA594037, SRR10674595–SRR10674614. The scripts are available in the Supplemental File.
