# Supplementary figures and images for: Alternative splicing and thermosensitive expression of Dmrt1 during urogenital development in the painted turtle, Chrysemys picta
Source: PeerJ. 2020 Mar 19;8:e8639. doi: 10.7717/peerj.8639 (PMC7085901; doi:10.7717/peerj.8639)

**A**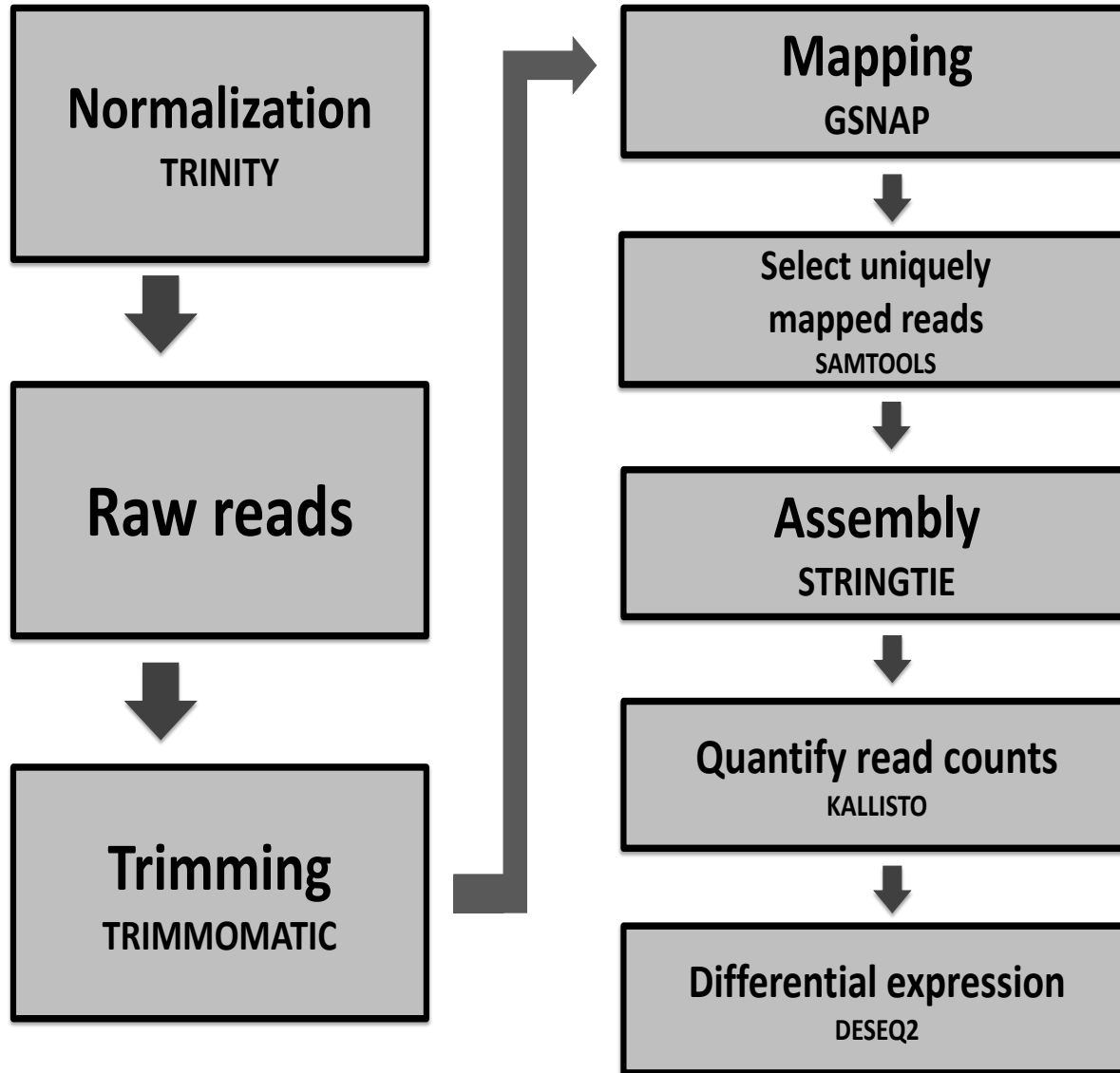**B**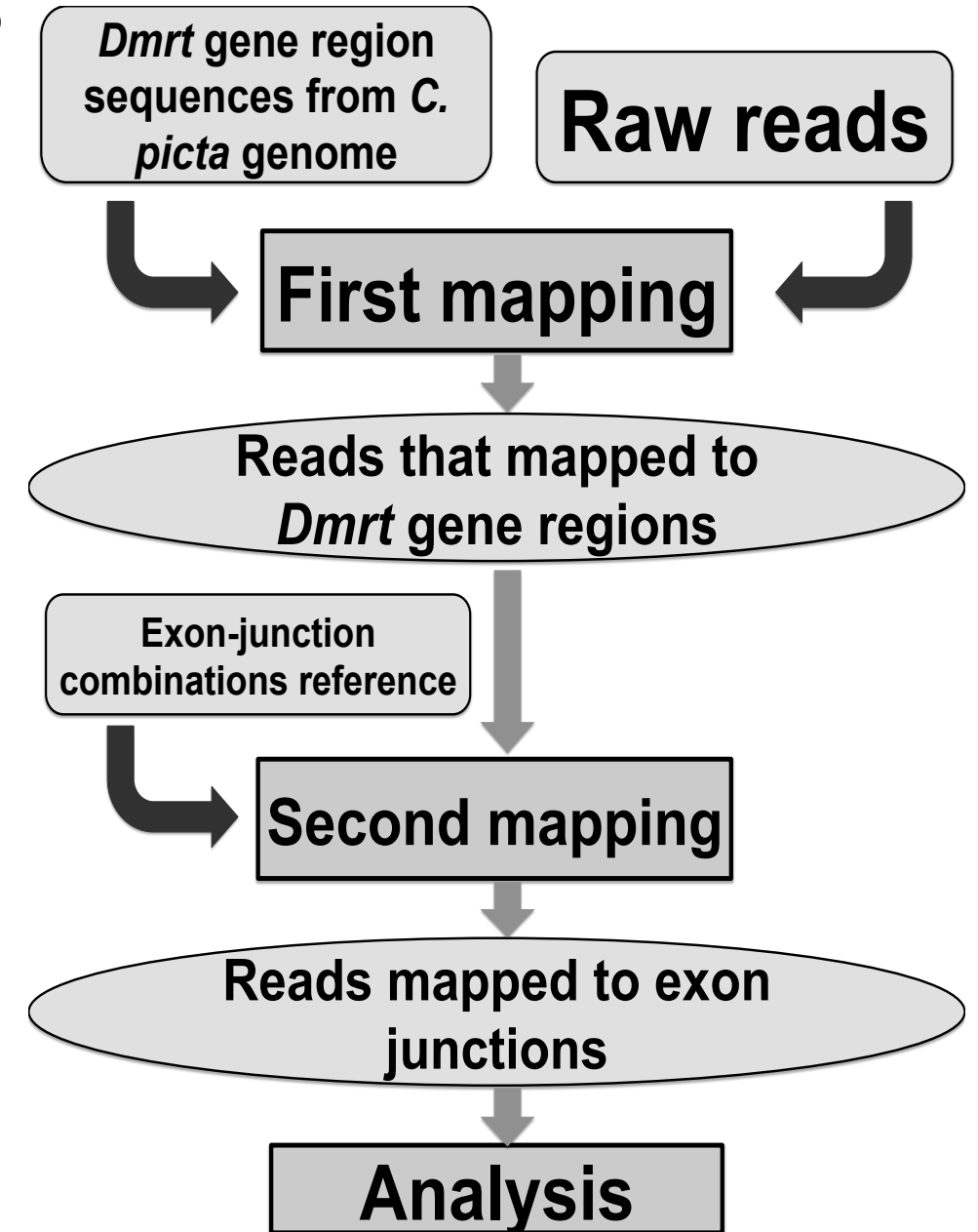

Supplement: Supplemental Information 2 [file peerj-08-8639-s002.pdf]

## A $\beta$ -actin transcript – RNA-Seq

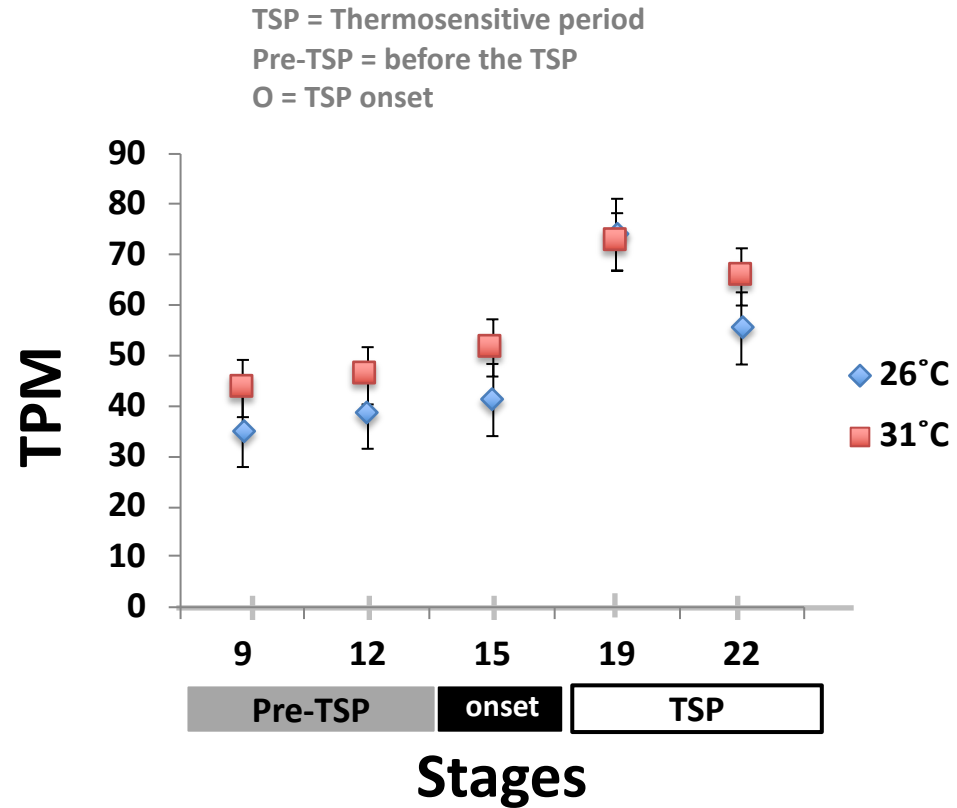

## B $\beta$ -actin transcript – qPCR

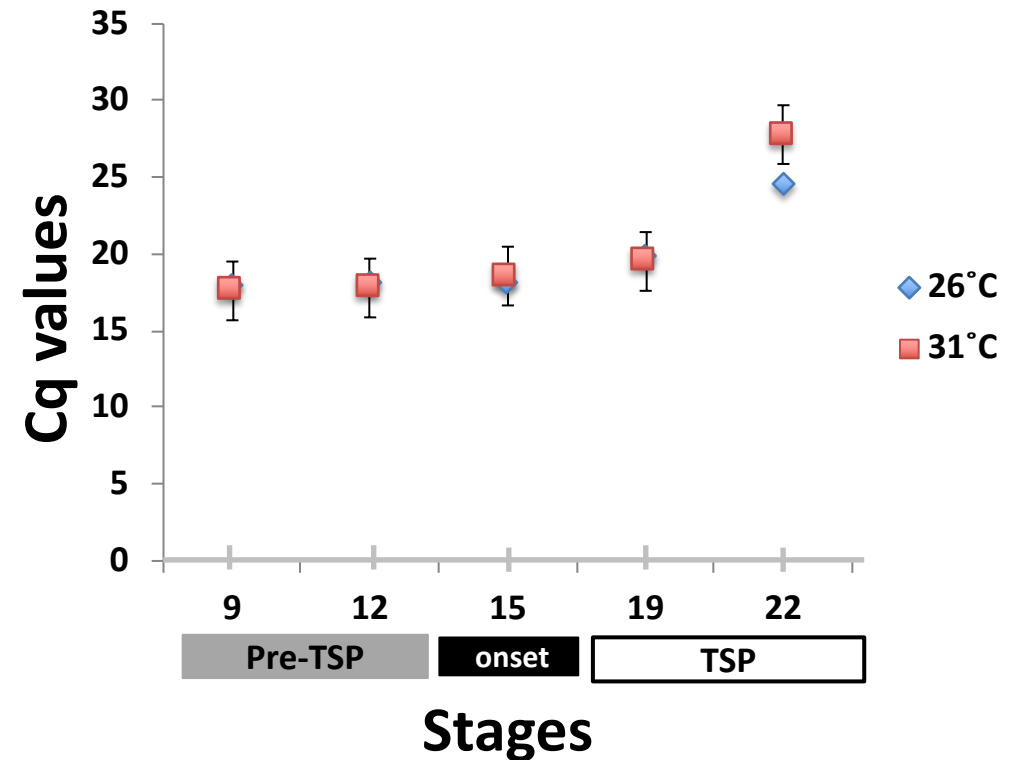

Supplement: Supplemental Information 5 — Panel A illustrates RNA-Seq results. Panel B illustrates TaqMan qPCR results. Bars represent standard deviations. [file peerj-08-8639-s005.pdf]
