# Supplementary material for: Alternative splicing and thermosensitive expression of Dmrt1 during urogenital development in the painted turtle, Chrysemys picta
Source: PeerJ. 2020 Mar 19;8:e8639. doi: 10.7717/peerj.8639 (PMC7085901; doi:10.7717/peerj.8639)
Supplement: Supplemental Information 3 [file peerj-08-8639-s003.pdf]

Scripts used in this study.

Locations and file names were substituted for data protection.

- Trimming - Trimmomatic

```
$ java -jar /path/to/Trimmomatic-0.36/trimmomatic-0.36.jar PE -phred33 read1.fastq
read2.fastq output-paired1.fq output-unpaired1.fq output-paired2.fq output-unpaired2.fq
LEADING:3 TRAILING:3 SLIDINGWINDOW:4:15 MINLEN:30
```

- Normalization - Trinity

```
$ /opt/rit/spack-app/linux-rhel7-x86_64/gcc-4.8.5/trinity-2.6.6-
nl7v7dqj3ket2r2wmo4uh4e7fkwzcuod/bin/util/insilico_read_normalization.pl --seqType fq
--JM 100G --max_cov 30 --left $fq1 --right $fq2 --pairs_together --PARALLEL_STATS --
CPU 16 --output normalized_reads
```

- Mapping - GSNAP

- build genome index

```
$ gmap_build -d genome-database-name -D /path/tp/genome/folder
/path/to/genome/file.fasta
```

- mapping

```
$ fq1=/path/to/normalized/Reads1
$ fq2=/path/to/normalized/Reads2

$ dir=/path/to/database #needs to be identical to what you specified when building
database
$ db=<name of database>

$ gsnap -D $dir -d $db -N 1 -t 16 -m 7 -A sam --pairexpect 300 -o out.sam $fq1 $fq2
#order of -D and -d is very important #-N option turns on novel splicing feature #-m is a
parameter for mismatches
```

- Mapping processing – Samtools

```
$ samtools view -bS out.sam #convert file to BAM format
$ samtools flagstat out.bam > out.stat.out #gives out statistics of mapping process
$ samtools sort  out.bam -o out.sorted.bam #sort files
```

- Assembly – StringTie

```
$ stringtie /path/to/sorted.bam -o output-assemble.gtf -c 1 -M 1 -C T26-S9-R1-
readcoverage.gtf -G /path/to/genome/annoation/file.gff3
$ stringtie --merge -p 8 -G /path/to/genome/annotation.gff3 -o output-merged.gtf
merge.txt #merge.txt is a text file that lists all assembly files
```

- Read counts – Kallisto

- Convert transcriptome gif file to fasta file – Cufflinks

```
$ gffread -w transcripts-output.fa -g /path/to/genome/file.fasta path/to/ output-
assemble.gtf
```

- Build index – Kallisto

```
$ kallisto index -i transcripts-output.idx transcripts-output.fa
```

- Quantify abundances

```
$ fq1=/path/to/normalized/Reads1
$ fq2=/path/to/normalized/Reads2

$ kallisto quant -i transcripts-output.idx -o kallisto-output-folder-name -b 100 $fq1 $fq2
```

- Differential expression analysis – DESEQ2

Following steps to be run on R

- Import Kallisto read count tables into R – tximport

```
if (!requireNamespace("BiocManager", quietly = TRUE))
  install.packages("BiocManager")

BiocManager::install("tximport")
BiocManager::install("rhdf5")
BiocManager::install("AnnotationDbi")
BiocManager::install("GenomicFeatures")
BiocManager::install("rtracklayer")
BiocManager::install("RMariaDB")
library(tximport)
library(readr)
library(AnnotationDbi)
library(GenomicFeatures)
library(rtracklayer)
library(RMariaDB)

#determine a matrix o samples informations
samples <- read.table("samples.txt", header = TRUE) #samples.txt is a txt file with a list
of folders to be imported

#import files into R
files <- file.path("/path/to/directory/with/kallisto/results", samples$run, "abundance.tsv")
names(files) <- paste0("sample", 1:4) #name each sample
head(files)
file.exists(files) #all need to be TRUE
```

```
#produce count matrices with annotations
txdb <- makeTxDbFromGFF("/path/to/genome/annotations.gff3")
seqinfo(txdb)
genes(txdb)
k <- keys(txdb, keytype = "TXNAME")
tx2gene <- select(txdb, k, "GENEID", "TXNAME")
head(tx2gene)
tail(tx2gene)

#Replacing NAs as zero so the data frame is readable
BiocManager::install("tidyr")
library(tidyr)
tx2genedm <- replace_na(tx2gene, list(TXNAME=0, GENEID=0))
head (tx2genedm)

#import file
txi <- tximport(files, type="kallisto", tx2gene = tx2genedm, txOut = TRUE, geneIdCol =
TRUE, abundanceCol = TRUE, countsCol = TRUE)
```

- Differential expression analysis

```
library("DESeq2")
ddsTxi <- DESeqDataSetFromTximport(txi,
                                   colData = samples,
                                   design = ~ temp)

#Run the default analysis for DESeq2 and generate results table
dds <- DESeq(ddsTxi9)
res <- results(dds9)
write.table(x = res, file = "DESEQ2-KALLISTO.csv")

counts <- counts(dds, normalized = TRUE)
write.csv(counts, file="norm_counts.csv")
```
