# Supplementary material for: Alternative splicing and thermosensitive expression of Dmrt1 during urogenital development in the painted turtle, Chrysemys picta
Source: PeerJ. 2020 Mar 19;8:e8639. doi: 10.7717/peerj.8639 (PMC7085901; doi:10.7717/peerj.8639)
Supplement: Supplemental Information 4 — Reads are separated by developmental stage and incubation temperature (26 °C = Male Producing Temperature, 31 °C = Female Producing Temperature). [file peerj-08-8639-s004.pdf]

Tables of read counts obtained from Kallisto. Reads are separated by developmental stage and incubation temperature (26˚C = Male Producing Temperature, 31˚C = Female Producing Temperature).

Table 1 – Read counts for the reference gene, ß-actin

ACTB	26˚C	31˚C
Stage 9	119.7537885	148.2066378
Stage 12	85.31827286	87.27149015
Stage 15	81.92285147	112.9892575
Stage 19	91.26996659	124.45333
Stage 22	76.58841836	74.78698857

Table 2 – Read counts for the *Dmrt1* canonical transcript

Dmrt1 canonical	26˚C	31˚C
Stage 9	2.422649092	2.161248462
Stage 12	0	1.045890836
Stage 15	2.418880734	0
Stage 19	49.85323249	0
Stage 22	123.5170211	6.575945712

Table 3 – Read counts for the *Dmrt1* ΔEx2Ex3 transcript

Dmrt1 ΔEx2Ex3	26˚C	31˚C
Stage 9	4.670833627	2.130633487
Stage 12	1.449632071	0.480599649
Stage 15	0.987823425	0
Stage 19	59.06395916	23.71100978
Stage 22	92.4728508	11.86918086
