# Supplementary material for: Alternative splicing and thermosensitive expression of Dmrt1 during urogenital development in the painted turtle, Chrysemys picta
Source: PeerJ. 2020 Mar 19;8:e8639. doi: 10.7717/peerj.8639 (PMC7085901; doi:10.7717/peerj.8639)
Supplement: Supplemental Information 1 — Sry is present only in Therian mammals. Modified from (Mizoguchi & Valenzuela, 2016) [file peerj-08-8639-s001.pdf]

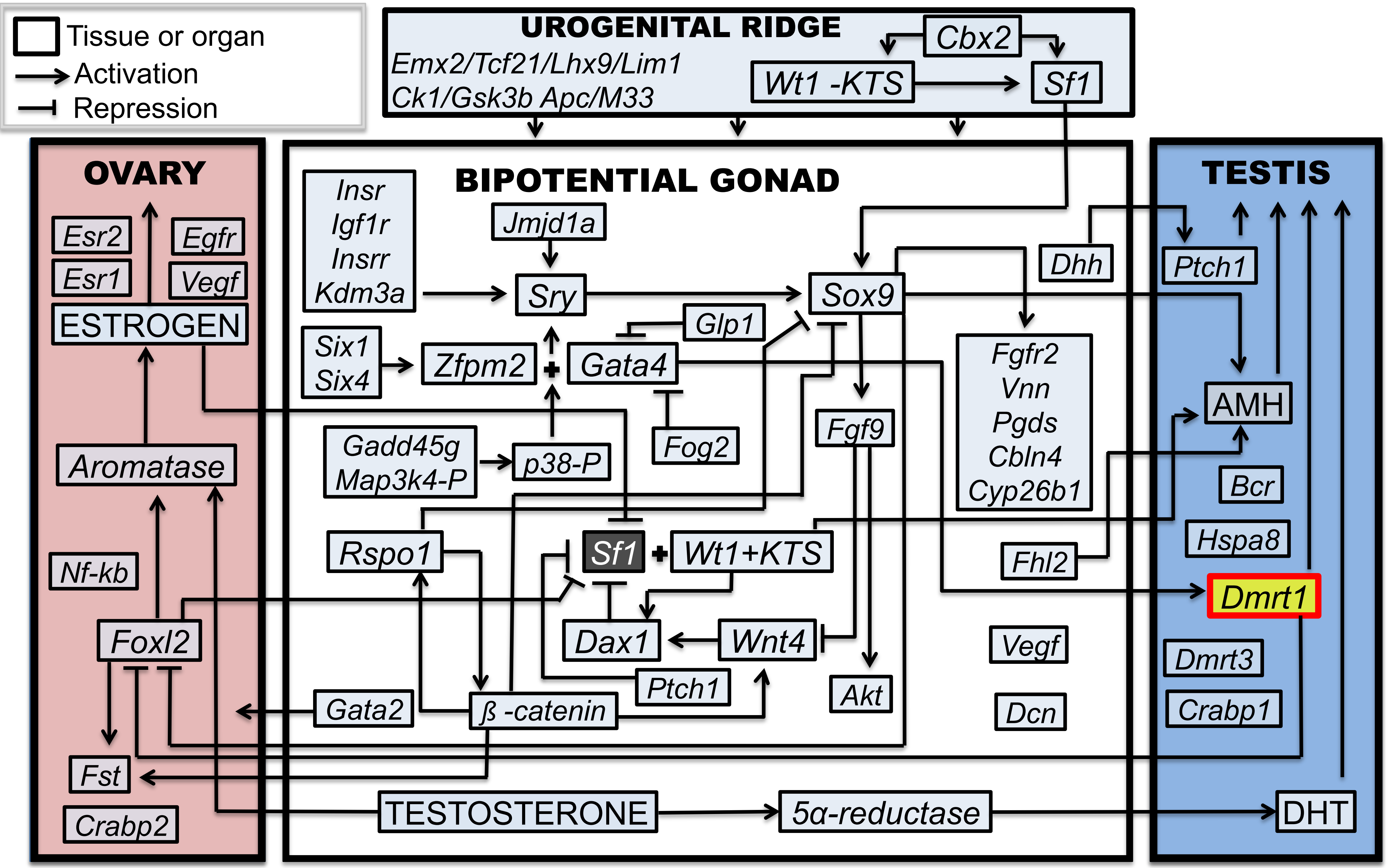

Tissue or organ
Activation
Repression
UROGENITAL RIDGE
Emx2/Tcf21/Lhx9/Lim1
Ck1/Gsk3b Apc/M33
Cbx2
Wt1 -KTS
Sf1
OVARY
Esr2
Egfr
Esr1
Vegf
ESTROGEN
Aromatase
Nf-kb
Foxl2
Fst
Crabp2
BIPOTENTIAL GONAD
Insr
Igf1r
Insrr
Kdm3a
Jmjd1a
Sry
Sox9
Dhh
Glp1
Six1
Six4
Zfpm2
Gata4
Fgfr2
Vnn
Pgds
Cbln4
Cyp26b1
Gadd45g
Map3k4-P
p38-P
Fog2
Fgf9
Rspo1
Sf1
Wt1+KTS
Fhl2
Dax1
Wnt4
Vegf
Ptch1
Akt
Gata2
ß -catenin
Dcn
TESTOSTERONE
5α-reductase
TESTIS
Ptch1
AMH
Bcr
Hspa8
Dmrt1
Dmrt3
Crabp1
DHT
